# Navigating nature’s toll: Assessing the ecological impact of the refugee crisis in Cox’s Bazar, Bangladesh

**DOI:** 10.1016/j.heliyon.2023.e18255

**Published:** 2023-07-13

**Authors:** Showmitra Kumar Sarkar, Mustafa Saroar, Tanmoy Chakraborty

**Affiliations:** Department of Urban and Regional Planning, Khulna University of Engineering & Technology (KUET), Khulna, 9203, Bangladesh

**Keywords:** Ecosystem, Machine learning, LULC, Ecology, Rohingya refugees

## Abstract

The Rohingya crisis in Myanmar’s Rakhine state has resulted in a significant influx of refugees into Cox’s Bazar, Bangladesh. However, the ecological impact of this migration has received limited attention in research. This study aimed to address this gap by utilizing remote sensing data and machine learning techniques to model the ecological quality (EQ) of the region before and after the refugee influx. To quantify changes in land use and land cover (LULC), three supervised machine learning classification methods, namely artificial neural networks (ANN), support vector machines (SVM), and random forests (RF), were applied. The most accurate LULC maps obtained from these methods were then used to assess changes in ecosystem service valuation and function resulting from the land use changes. Furthermore, fuzzy logic models were employed to examine the EQ conditions before and after the Rohingya influx. The findings of the study indicate that the increased number of Rohingya refugees has led to a 9.58% decrease in forest area, accompanied by an 8.25% increase in settlement areas. The estimated total ecosystem services value (ESV) in the research area was $67.83 million in 2017 and $67.78 million in 2021, respectively. The ESV for forests experienced a significant decline of 21.97%, equivalent to a decrease of $5.33 million. Additionally, the reduction in forest lands has contributed to a 13.58% decline in raw materials and a 14.57% decline in biodiversity. Furthermore, utilizing a Markovian transition probability model, our analysis reveals that the EQ conditions in the area have deteriorated from “very good” or “good” to “bad” or “very bad” following the Rohingya influx. The findings of this study emphasize the importance of integrating ecological considerations into decision-making processes and developing proactive measures to mitigate the environmental impact of such large-scale migrations.

## 1 Introduction

The political violence in Myanmar's Rakhine state in 2017 triggered a massive influx of Rohingya refugees into neighboring Bangladesh [1]. The prefabricated shelters in Bangladesh's southern mountainous regions were established as part of the humanitarian response to this crisis. Previous studies have found a correlation between deforestation and the arrival of Rohingya migrants [[2], [3], [4]]. The refugees have cut down trees, largely for constructing temporary shelters and for fuel [2]. Over 720,000 Rohingya refugees have fled Myanmar since August 2017 and sought refuge in the congested settlement camps in the Ukhiya sub-district of Rakhine state [5]. The massive influx of refugees into the forested regions of Ukhiya has threatened natural and planted forests, as well as social forestry programs [6]. This has caused ecological concerns for both the displaced Rohingya community and the host community [7].

The camp expansion has officially caused damage to approximately 2000 ha of forest after the arrival of over 750,000 Rohingya refugees in August 2017 [8]. The 2500 ha of land “lost” to Rohingya camps was estimated to be worth BDT 741.31 billion (c. US$86.67 million) in 2018 [9]. Research has shown that deforestation caused by human activities has negative impacts on the environment, including but not limited to: destruction of wildlife habitat; soil erosion and desertification; disruption of the water cycle; loss of traditional livelihoods; and increased ecological risks due to forest fragmentation [10,11]. The refugee influx has also led to environmental threats such as pollution, depletion of subsurface water supplies, habitat loss, and fragmentation, which have resulted in the extinction of threatened wild forest species.

Studies are being conducted to examine the potential impact of Rohingya refugees on various types of land cover (see, for example, [[12], [13], [14], [15]]). Researchers, such as [2,3,[16], [17], [18]], believe that the influx of Rohingya refugees and changes in forest cover are interconnected. The functioning of ecosystem services in the region affected by the Rohingya refugee crisis is impacted by changes in land use/land cover (LULC) [4,[19], [20], [21]]. Furthermore, several studies have linked the Rohingya refugee influx to above-ground biomass [13], socioeconomic and environmental conditions [22], land surface temperature (LST) in the Rohingya camp region [3,23], and human-elephant conflicts [24], food prices [25], host community’s relations to places [26], soil moisture, and evapotranspiration [27]. However, no research has yet attempted to quantify the actual rate of ecological quality (EQ) degradation due to the Rohingya refugee influx. This study aims to fill this gap by evaluating EQ conditions using human population, ecosystem services, and environmental factors from before and after the Rohingya refugee influx. This novel research approach represents a data-driven evaluation of EQ through the use of machine learning and fuzzy logic models. To the best of the authors' knowledge, this is the first study to use this novel method to assess changes in EQ in the region with the largest refugee influx. The study is not a policy document, but it will help the policy maker make decisions about conservation strategies, priorities, and public policies aimed at saving an ecologically vulnerable areas.

The study applied three machine learning methods to quantify settlement growth and forest cover loss in the Ukhiya and Teknaf regions of southern Bangladesh. A quantitative approach was taken to estimate the values of ecosystem services in response to changes in LULC patterns. Furthermore, land use-specific carbon emissions and absorption, as well as land surface temperature (LST), were estimated as inputs for the model. Finally, fuzzy logic models were used to estimate EQ conditions before and after the refugee influx. As far as the authors are aware, this is the first study to estimate the effects of the Rohingya refugee influx on ecosystems and EQ conditions, and the combination of machine learning and fuzzy logic models for estimating the impact on natural resource availability, ecosystem services, and ecological quality is a new approach. The research aims to achieve the following specific objectives: (1) to investigate the changes in LULC patterns before and after the influx of the Rohingya influx; (2) to estimate the impact of the observed land use changes on the valuation and functioning of ecosystem services, considering the changes that occurred both before and after the Rohingya influx; and (3) to develop a robust ecological quality condition model using fuzzy logic, allowing for a comprehensive assessment of ecological conditions before and after the Rohingya influx, thereby providing valuable insights into the changes in environmental health and sustainability.

The study aims to help policy makers make informed decisions about conservation strategies, priorities, and public policies aimed at saving ecologically vulnerable areas affected by the influx of Rohingya refugees. By assessing the ecological quality (EQ) condition using data-driven methods, the study provides insight into the impacts of the Rohingya refugee influx on land use/land cover, ecosystem services, and environmental factors. This information can be used to develop and implement management strategies to mitigate negative impacts and promote sustainability in the region. The use of machine learning algorithms and statistical techniques allows for the mapping and quantification of changes in land use and land cover, estimation of changes in ecosystem service values, and building of an EQ model. This novel approach to investigating EQ can be valuable in proposing effective management strategies to conserve the environment and its resources.

## 2 Materials and methods

### 2.1 Description of the study area

**Fig. 1:**
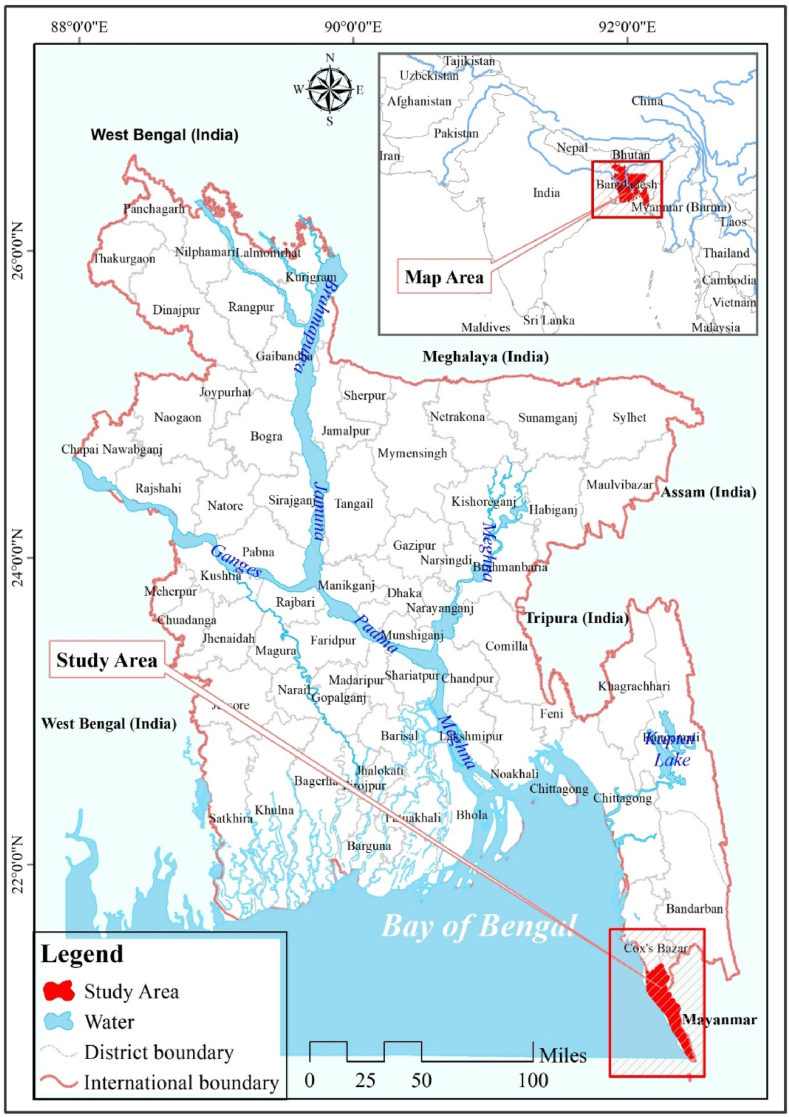
Study area.

Bangladesh is at risk from many natural disasters [28,29]. The Cox’s Bazar district and surrounding coastal areas in southeast Bangladesh are particularly vulnerable to the impacts of Rohingya immigration [30]. The majority of Rohingya refugees arrived in Bangladesh in 1991, 2012, and 2017, with a total of 914,998 refugees present as of September 30, 2019 (880,133 counted and 34,917 registered) [31]. The majority of refugee camps are located in Teknaf and Ukhiya upazilas in the Cox’s Bazar district, which is the focus of the study (Fig. 1). This region features a diverse range of landscapes, including rolling hills, flat piedmonts, tidal floodplains, and a 120-km stretch of sandy beach along the Bay of Bengal, which some consider to be the longest beach in the world. Partially wooded slopes, ranging from 100 to 250 m in height, line both shores. The Piedmont Plains, heavily populated by humans, are sloping lands in the foothills with a maximum elevation of 10 m. The 3155 ha of sandy coastline along the western shore of the peninsula account for 9.03% of its total land area.

### 2.2 Materials

In order to achieve the objectives of the research, information from both geographical and demographic sources was combined. High-resolution multispectral satellite images from Sentinel-2A and Sentinel-2B with a spatial resolution of 10 m in the visible and NIR bands were used to derive large-scale land cover information. Sentinel satellite imagery for the study area was gathered from two time periods: before the Rohingya refugee influx (August 25, 2017) and after it (March 20, 2021). 600 random locations were selected as samples from WorldView-2 images with a spatial resolution of 0.5 m, and these sample sites were validated through field-based observations and surveys. The sample locations were divided into two groups: a training set consisting of 80% of the samples (480 samples) and a testing set consisting of 20% of the samples (120 samples). The sample locations were categorized into four groups (agriculture, forest, settlement, and water) based on different levels of land use and land cover. Changes in land surface temperature were estimated using two time series (2017 and 2021) of Landsat 8 Operational Land Imager (OLI) images (path/row 139/43) obtained from the USGS website (https://earthexplorer.usgs.gov).

### 2.3 Methods for land use land cover map

Land use and land cover classification is a common practice in remote sensing studies. As per previous research, three machine learning methods were used to achieve accurate results, and the best one was selected based on overall accuracy and Kappa coefficient. The following section discusses the mapping methods for three land use and land cover types.

#### 2.3.1 Artificial Neural Network (ANN)

An Artificial Neural Network (ANN) is a type of machine learning algorithm used in artificial intelligence. It is composed of input, hidden, and output layers and works by computing, learning, and error correction [32]. The learning process is slow and imperceptible, and the parameters such as training rate, training momentum, training root-mean-square error exit threshold, and the number of training iterations influence the output. In this study, the number of training iterations was set to 1000, as suggested by Ref. [33].

#### 2.3.2 Support Vector Machine (SVM)

Support Vector Machine (SVM) is an algorithm commonly used in satellite data classification and known for its ability to separate interclass hyperplanes effectively [34]. In cases of non-linear class separation, a soft margin approach is used, and the regularization constant (cost) is applied to minimize errors. The radial basis function (RBF) kernel-based SVM method was used in this study, and the optimal C parameter was determined by mapping the LULC using various values, as described by Ref. [35].

#### 2.3.3 Random Forest (RF)

Random Forest (RF) is a machine learning classifier that uses an ensemble learning strategy and combines multiple tree predictors. As the number of trees increases, the generalization error converges, and overtraining is not a problem [36]. In most studies, the default settings provide sufficient results, and using more trees than necessary does not hurt the model [37,38]. In this study, the optimal RF model for classification was determined by looking at a wide range of values for the mtry and ntree parameters, with ntree being set to 500 and mtry to 5.

### 2.4 Validation of the LULC maps and selection of best LULC map

Verifying the precision of a LULC classification result is crucial before it can be utilized in practice. Three LULC maps were generated for both the pre- and post-Rohingya influx using machine learning algorithms. The accuracy of these maps was measured using the Kappa coefficient and the overall accuracy (OA). As stated by Ref. [39], investigating the sources of confusion in the confusion matrix is an essential step in developing a map from remotely sensed data. To evaluate the classification accuracy of a map, a confusion matrix is constructed to compare reference and labeled pixels. The results from the Kappa test will determine the best LULC model to use.

### 2.5 Estimation of ESV

There are various approaches for valuing ESV estimation in monetary units [40]. identified four main techniques, including the expressed preference method, the revealed preference method, the cost-based method, and the benefit transfer method. The benefit transfer method (BTM) has become popular due to its practicality and simplicity, as noted by Refs. [[41], [42], [43]]. This method allows for the estimation of the value of each ecosystem service by considering the unique value it provides for a particular type of land cover [41,43]. found that this approach has been justified through over 300 case studies from around the world. In this analysis, the ESVs for 16 out of 17 ESs were estimated using the LULC. Agriculture, forests, settlements, and water in the research region were matched to farmland, forests, urban areas, and wetlands, respectively, using the ESs model of [42] (Table 1). The ESV was determined using equations (1), (2), (3) as suggested by Ref. [44].

(1) $${ESV}_{k}=\sum_{f}A_{k}\times {VC}_{kf}$$

(2) $${ESV}_{f}=\sum_{k}A_{k}\times {VC}_{kf}$$

(3) $${ESV}_{f}=\sum_{f}\sum_{k}A_{k}\times {VC}_{kf}$$

**Table 1:** Coefficients of different LULC categories for estimating ESV (US$/ha/year).

ES type	Sub-type	Agriculture	Forest	Settlement	Water
Provisioning	Food production	54	43	0	41
	Raw material	0	138	0	0
Regulating	Gas regulation	0	0	0	0
	Climate regulation	0	141	0	0
	Disturbance regulation	0	2	0	0
	Water regulation	0	2	0	5445
	Water supply	0	3	0	2117
	Waste treatment	0	87	0	665
Supporting	Soil-formation and retention	0	10	0	0
	Nutrient cycling	0	361	0	0
	Erosion control	0	96	0	0
	Pollination	14	0	0	0
	Biodiversity	24	2	0	0
	Genetic resources	0	16	0	0
Culture	Recreation and tourism	0	68	0	230
	Cultural	0	2	0	0

ESV_k_ = Ecosystem Service Value for LULC Type k, A_k_ = Area for LULC Type k in Hectares, VC_fk_ = Value Coefficient for Function f for LULC Type k in US Dollars per Hectare per Year, ESV_f_ = Ecosystem Service Value for Service Function f, ESV = Total Ecosystem Service Value.

To identify the uncertainties of ESV assessment, sensitivity analysis was conducted to estimate the changes in ESV in response to 50% adjustments of the ESV coefficients for each LULC type [45]. Coefficient of sensitivity (CS) was calculated using equation (4), which is based on the basic economic notion of elasticity [44].

(4) $$CS=\frac{\left({ESV}_{j}-{ESV}_{i}\right)/{ESV}_{i}}{\left({VC}_{jk}-{VC}_{ik}\right)/{VC}_{ik}}$$

ESV and VC are ecosystem service value and coefficient value, respectively, for initial (i) and adjusted (j) situations. The k represents various LULC categories. According to CS value, the estimated ESV can be elastic (CS > 1) or inelastic (CS < 1).

### 2.6 Ecological quality (EQ) modeling with fuzzy logic

In the present study, ecological condition modeling was conducted for 2017 and 2021 using fuzzy logic in a series of steps.

Step 1: Derivation of Parameters

The ecological quality conditions were modeled based on seven parameters: human population including Rohingya, land surface temperature (LST), land use-specific carbon emissions and absorptions, provisioning, regulating, supporting, and cultural ecosystem services for 2017 and 2021. Four ecosystem function parameters were already created by integrating land use and land cover (LULC) and the coefficient of ecosystem functions, as proposed by Ref. [42], for both periods. Land-specific carbon emissions and absorptions were estimated for both periods based on the coefficients found in Ref. [46]. The population data, including the Rohingya influx, was integrated for both 2017 and 2021. LST was estimated for both years from Landsat 8 OLI images, which has become more common with the development of new methods, such as the mono-window approach [47] and the single-channel algorithm [48,49]. The mono-window technique used to derive LST from Landsat TIR data requires three factors: ground emissivity, atmospheric transmittance, and effective mean air temperature. The original TIR bands were resampled from 120 m, 60 m, and 100 m–30 m for further analysis.

Step 2: Modeling Process

After determining the parameters for both periods, fuzzy logic was used to construct the EQ condition. The fuzzy membership function was used on the parameters to make them fuzzy crisp. The degree of membership function with the target is shown by the fuzzy-crisp layers, where values of any fuzzy-crisp parameter vary from 0 to 1, with 0 indicating a very low fuzzy membership function with the target and 1 indicating a very high fuzzy membership function with the target. A ‘small’ membership function was used to create fuzzy-crisp layers for the four ecosystem functions, as a low value of ecosystem function may significantly affect EQ. A ‘large’ membership function was selected for population, LST, and land-specific carbon emissions and absorptions, as high values of these variables significantly affect the EQ condition. All parameters were made direction-free, such that a high value of any variable can have a significant impact on EQ, with a high value indicating a deteriorating EQ condition and vice versa.

Fuzzy operators, such as ‘AND’, ‘OR’, and ‘GAMMA’, were used after converting all parameters to fuzzy-crisp layers. The models were visualized, and it was found that the GAMMA model performed best. The best-performing model was created using a GAMMA coefficient of 0.9. The same process was then repeated for the EQ conditions in 2021.

Step 3: Change Vector Analysis

In this work, a Markovian transition matrix and an alluvial diagram were used to investigate the changes in the environmental evaluation caused by the influx of Rohingya refugees in Bangladesh. The Markovian transition matrix provides a rough estimate of the number of pixels that were converted from one LULC type to another over the chosen time period. The heat map was designed to depict the Markovian transition matrix between the 2017 and 2021 EQ models. The following matrix depicts these probabilities visually:

$$p=pij=\left(\begin{array}{ccc} p_{11} & p_{12} & p_{1m}\\ p_{21} & p_{22} & p_{2m}\\ p_{31} & p_{32} & p_{3m} \end{array}\right)$$

where, p = the state of probability of transition from i to j.

**Fig. 2:**
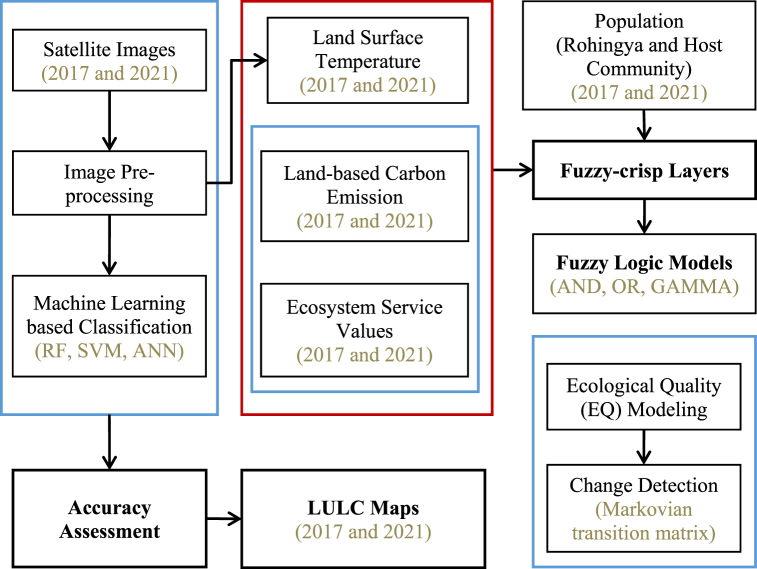
Methodological framework of the study.

In addition, an alluvial plot was used to show better the transition between the 2017 and 2021 EQ models. The overall methodological framework of the research is shown in Fig. 2.

## 3 Results

### 3.1 Impact of Rohingya refugee influx on different LULC

**Fig. 3:**
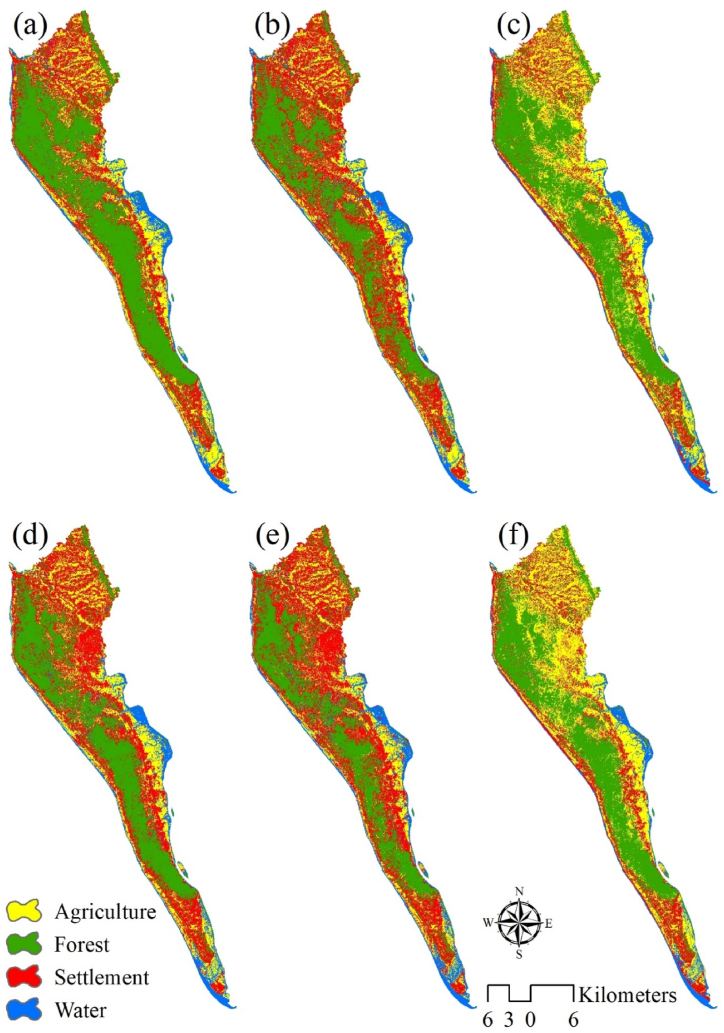
Spatial distribution of LULC using (a) ANN, (b) RF, and (c) SVM in 2017 (i.e., before Rohingya Refugee Influx) and (d) ANN, (e) RF, and (f) SVM in 2021 of Ukhiya and Teknaf Upazilas.

The impact of the Rohingya refugee influx on different LULC has been analyzed through image classification techniques in 2017 and 2021 (Fig. 3(a–f)). The classification was performed using three algorithms: RF, SVM, and ANN. The results showed that the ANN model had the highest accuracy, with kappa statistics of 0.94, 0.86, and 0.96 for RF, SVM, and ANN, respectively in 2017. In 2021, the kappa statistics were 0.87, 0.81, and 0.89 for RF, SVM, and ANN, respectively. The overall accuracy of the ANN model was found to be 0.97 in 2017 and 0.91 in 2021 (Table 2).

**Table 2:** The accuracy assessment of LULC mapping using machine learning algorithms.

Machine learning algorithms	Kappa statistics		Overall accuracy	
	2017	2021	2017	2021
Artificial Neural Network	0.96	0.89	0.97	0.91
Random Forest	0.94	0.87	0.95	0.90
Support Vector Machine	0.86	0.81	0.89	0.86

Before the refugee influx in 2017, the study region had different LULCs, with 43.59% of the area covered by forests, 27.25% by inhabited areas, 20.44% by farms and other agricultural uses, and 8.72% by water bodies (Table 3 and Fig. 3(a)). After the influx in 2021, the area covered by settlements increased to 35.49%, followed by 34.02% by forests, and 20.68% by agricultural lands. The water bodies in the study region decreased to 9.81% (Table 3 and Fig. 3(d)).

**Table 3:** Area changes of Land Use/Land Cover in Ukhiya and Teknaf Upazilas from 2017 to 2021.

Land Use/Land Cover	2017		2021		Change	
	Area [ha]	Percentage	Area [ha]	Percentage	[ha]	Percentage
Agriculture	11711.33	20.44	11853.41	20.68	142.08	0.25
Forest	24982.31	43.59	19494.3	34.02	−5488.01	−9.58
Settlement	15,614	27.25	20339.45	35.49	4725.45	8.25
Water	4999.61	8.72	5620.09	9.81	620.48	1.08
Total	57307.25	100	57307.25	100		

The increased settlement in the study area has led to the conversion of 5488 ha of forest cover into settlements, causing a reduction of 9.58% in the total forest cover. This conversion has affected the environment, livelihood, and biodiversity in the study area [4]. The conversion of some forest cover and bodies of water into agriculture has also been observed.

### 3.2 Impact of Rohingya refugee influx on total ESV

The estimated values for ecosystem services (ESV) in the research region are presented in Table 4 for various time intervals. In 2017, the total ESV was estimated to be $67.83 million, with water accounting for 62.64% of that value, forests contributing 35.76%, farms 2.65%, and cities 0%. In 2021, the projected ESV for the research area was $67.78 million, with the majority of the value coming from water (70.46%, or $47.76 million), followed by forests (27.19%) and agriculture (1.61%). Between 2017 and 2021, the ESV for water increased by 12.41%, while the ESV for agriculture rose by 1.21%. During the same period, the ESV for forests decreased by 21.97%, or $5.33 million.

**Table 4:** Ecosystem service value of Ukhiya and Teknaf Upazilas from 2017 to 2021.

LULC types	Ecosystem service value (million US$)		Changes from 2017 to 2021	
	2017	2021	million US$	percentage
Agriculture	1.08	1.09	0.01	1.21
Forest	24.26	18.93	−5.33	−21.97
Settlement	0.00	0.00	0.00	0.00
Water	42.49	47.76	5.27	12.41

Based on the sensitivity analysis conducted in the study, it was found that the CS values for agriculture, forest, and water in both 2017 and 2021 were less than 1. Importantly, all estimated ESV exhibited inelasticity with respect to the ecosystem value coefficients. The inelasticity of the estimated ESV indicates that changes in the ecosystem value coefficients had a relatively minor impact on the overall ESV. This finding suggests that the estimated ESV were highly precise and reliable.

### 3.3 Impact of Rohingya refugee influx on ES functions

**Fig. 4:**
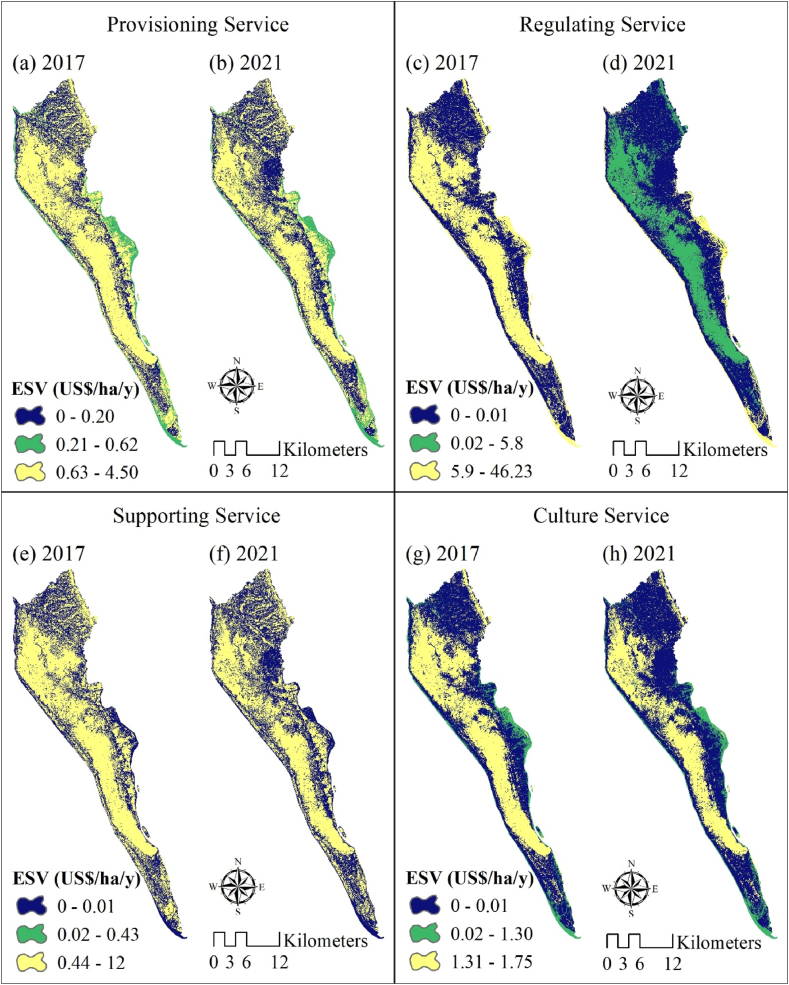
Spatial distribution of ecosystem service values: (a) provisioning, (c) regulating, (e) supporting, and (g) culture in 2017 (i.e., before Rohingya Refugee Influx) and (b) provisioning, (d) regulating, (f) supporting, and (h) culture in 2021 of Ukhiya and Teknaf Upazilas.

Regarding the impact of the Rohingya refugee influx on the ES functions, Table 5 presents the expected ESV for each ES function. In 2017, the largest contribution to the ESV was from regulatory agencies ($47 million), followed by supporting services ($12.56 million), supplying services ($5.36 million), and cultural services ($2.9 million). By 2021, the expected ESV for regulatory services is expected to increase significantly, to $50.82 million, while the expected contributions from supporting, supplying, and cultural services are anticipated to decline, to $9.9 million, $4.4 million, and $2.66 million, respectively. Between 2017 and 2021, the ESV for 12 sub-functions decreased, while the ESV for water regulation, water supply, and pollination increased by 12.35%, 12.17%, and 1.21%, respectively. The distribution of ESV across different ES purposes is illustrated in Fig. 4(a–h).

**Table 5:** Estimated values for different ecosystem service functions of Ukhiya and Teknaf Upazilas from 2017 to 2021.

Ecosystem functions	Sub-types	Ecosystem service value (million US$)		Changes from 2017 to 2021	
		2017	2021	million US$	percentage
Provisioning	Food production	1.91	1.71	−0.20	−10.61
	Raw material	3.45	2.69	−0.76	−21.97
Regulating	Gas regulation	0.00	0.00	0.00	0.00
	Climate regulation	3.52	2.75	−0.77	−21.97
	Disturbance regulation	0.05	0.04	−0.01	−21.97
	Water regulation	27.27	30.64	3.37	12.35
	Water supply	10.66	11.96	1.30	12.17
	Waste treatment	5.50	5.43	−0.06	−1.18
Supporting	Soil-formation and retention	0.25	0.19	−0.05	−21.97
	Nutrient cycling	9.02	7.04	−1.98	−21.97
	Erosion control	2.40	1.87	−0.53	−21.97
	Pollination	0.16	0.17	0.00	1.21
	Biodiversity	0.33	0.32	−0.01	−2.29
	Genetic resources	0.40	0.31	−0.09	−21.97
Culture	Recreation and tourism	2.85	2.62	−0.23	−8.09
	Cultural	0.05	0.04	−0.01	−21.97

### 3.4 EQ condition assessment before and after Rohingya refugee influx

Seven variables were considered in the assessment of ecosystem quality (EQ) for the years 2017 and 2021, ranging from climate to population pressure and ecosystem functions such as food supply and recreation. The description of the ecosystem functions, including land surface temperature (LST), carbon emission and absorption, and population, has already been provided in the previous section.

The LST and carbon emission and absorption data were then transformed into a fuzzy-crisp layer, which ranges from 0 to 1, using the fuzzy membership function ‘large’. A value close to 1 indicates a high degree of membership and a higher deterioration of EQ conditions, while a value close to 0 represents the opposite.

Additionally, population growth was taken into consideration as a parameter in the EQ assessment for 2017 and 2021. The base population data from 2011 was obtained from the Census of Bangladesh, and the number of Rohingya refugees for 2017 and 2021 was integrated with this data. The minimum population was fixed as only the data of Rohingya refugees was added. The population data was also transformed into a fuzzy-crisp layer using the ‘large’ fuzzy membership function because a larger population can negatively impact the EQ conditions.

**Fig. 5:**
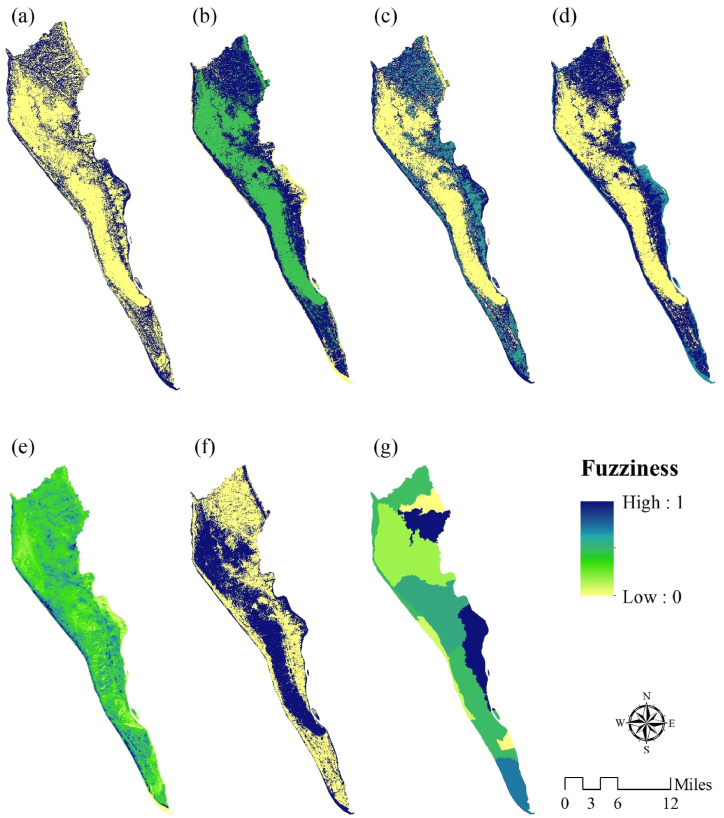
Parameters used for EQ assessment for 2017, such as: (a) fuzzy-crisp provisioning function, (b) fuzzy-crisp regulating function, (c) fuzzy-crisp supporting function, (d) fuzzy-crisp culture function, (e) fuzzy-crisp LST, (f) fuzzy-crisp land-use based carbon emission and absorption, (g) fuzzy-crisp population.

**Fig. 6:**
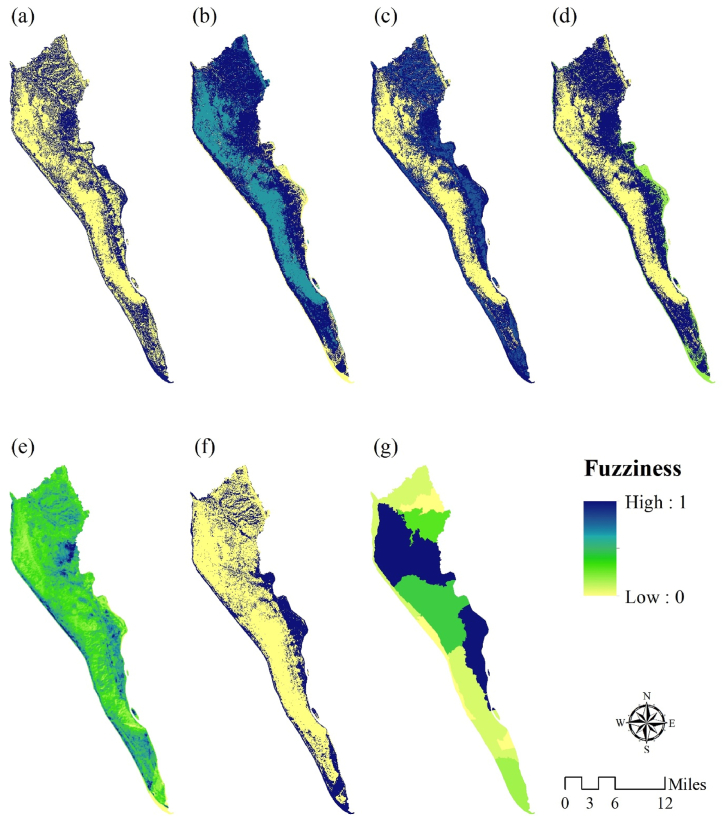
Parameters used for EQ assessment for 2021, such as: (a) fuzzy-crisp provisioning function, (b) fuzzy-crisp regulating function, (c) fuzzy-crisp supporting function, (d) fuzzy-crisp culture function, (e) fuzzy-crisp LST, (f) fuzzy-crisp land-use based carbon emission and absorption, (g) fuzzy-crisp population.

The ecosystem functions, including provisioning, regulating, supporting, and cultural functions, were represented as fuzzy-crisp layers for 2017. A high membership value indicates a worsening of EQ conditions. Fig. 5, Fig. 6 depict the parameters used in the EQ assessments for 2017 and 2021, respectively. The results indicate a decrease in the functioning capacity for all functions.

**Fig. 7:**
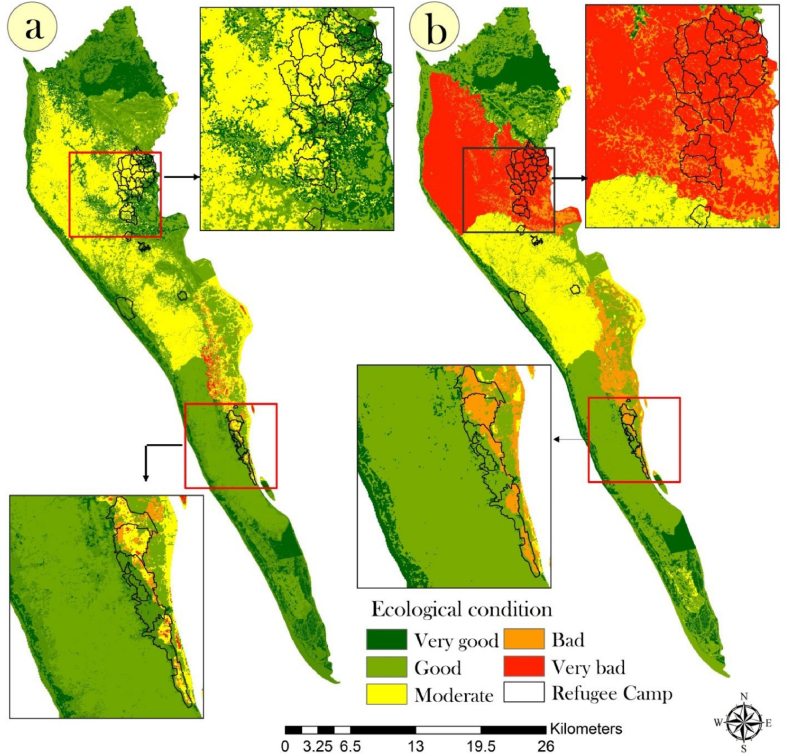
EQ assessments using fuzzy logic for (a) 2017 and (b) 2021.

This study integrated all fuzzy crisp layers using fuzzy operators to assess ecosystem quality (EQ) conditions in 2017 and 2021. The study used the GAMMA 0.9 operator to model EQ conditions, which performed better than other fuzzy operators. The EQ conditions were classified into five categories: very good, good, moderate, bad, and very bad. A “very poor” EQ condition indicates high levels of human disturbance, such as high temperatures and population pressure, which negatively impact ecosystem functioning. In contrast, a “very high” EQ condition indicates low levels of human disturbance and good ecosystem functioning. The study found that the influx of Rohingya refugees has led to increased deforestation in forest regions, causing the EQ conditions to worsen in these areas, disrupting both ecosystem functions and the climate. In 2017, only a few areas had poor EQ conditions, but these have been exacerbated by the increased influx of refugees in 2021 (Fig. 7(a–b)).

**Fig. 8:**
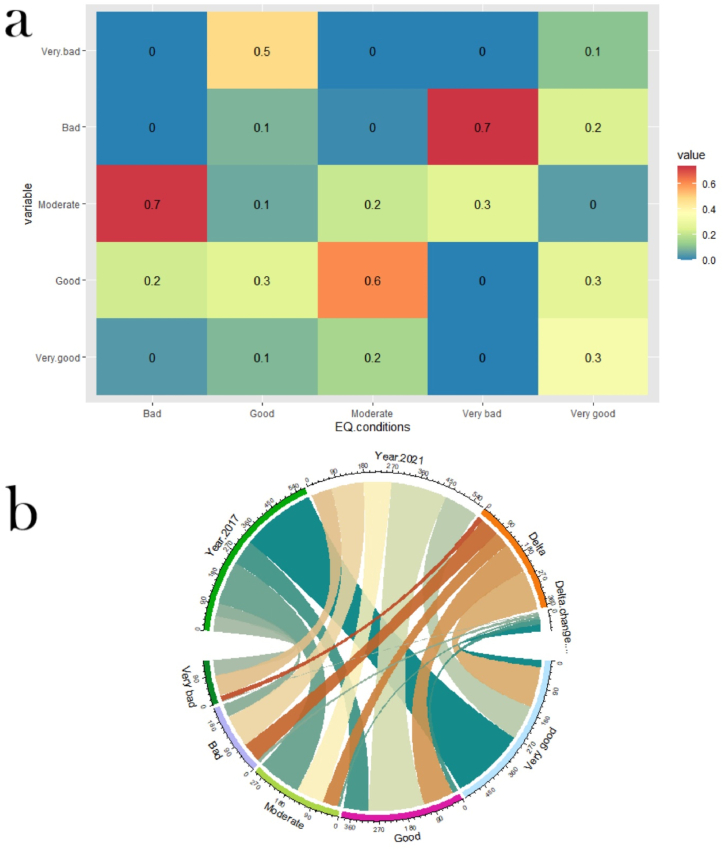
Change analysis of EQ condition between 2017 and 2021 using (a) Markovian transition matrix (x-axis represents EQ condition of 2017 and y-axis represent EQ condition of 2021), (b) area transition using alluvial plot.

The Markovian transition matrix between the EQ models of 2017 and 2021 was applied in this study, and the results were presented in a heat map (Fig. 8(a)). The values of the transition matrix indicate the probability of transitioning from one EQ class to another. Fig. 8(a) displays a 24.2% probability of transitioning from very good to bad or very bad EQ conditions, while the probability of transitioning from good EQ conditions to bad or very bad conditions is 9% and 48.68%, respectively. The transition probability from moderate EQ to good EQ is 61% over the four-year period, due to the removal and relocation of Rohingya refugee camps to Ratna Palong and Teknaf regions. However, this result may also have been impacted by the COVID-19 pandemic. On the other hand, bad EQ has a 73% probability of transitioning to moderate EQ, and very bad EQ has a 74% probability of transitioning to bad EQ, due to the new settlements for Rohingya refugees that have emerged and have high EQ.

To make the calculated area and delta change rate easier to understand, an alluvial plot was used (Fig. 8(b)). According to the findings, 133.67 km^2^ of areas with very good EQ transitioned to other EQ conditions, while areas with good EQ conditions gained 102.22 km^2^. Extremely poor and poor EQ conditions gained 18 km^2^ and 64.03 km^2^, respectively, while moderate EQ conditions lost 50.58 km^2^ of area. It can be concluded that areas with good EQ conditions increased because the relocation of Rohingya refugees led to the identification of new areas with bad and very bad EQ conditions.

## 4 Discussion

Ukhiya and Teknaf upazilas in Bangladesh hosted the majority of Rohingya refugees. The monitoring of environmental as well as social issues caused by the Rohingya refugee influx is highly recommended. Thus, this study was aimed at investigating the impact of the Rohingya refugee influx on ecological quality through remote sensing and machine learning algorithms. The impact of the massive Rohingya refugee inflow in 2017 has been felt most strongly in Ukhiya and Teknaf Uazilas, where the LULC has undergone fast transformation. Thousands of Rohingya people have fled political unrest in Rakhine State, Myanmar. As part of the humanitarian response, the government of Bangladesh has set up temporary shelters in the research areas for the Rohingya refugees who have crossed the Naf River, the majority of whom are women and children. The Rohingya have been coerced into migrating to Bangladesh since the 1970s, and the government there has always been welcoming. In 2017, hilly woodland made up 43.59% of the LULC in the research region (see Table 3). Because hilly forests have more public property than other LULC types, Rohingya refugees have occupied them. By cutting down forests and hills, Rohingya refugees have lived in clusters. With no baseline assessment, providing rapid shelters in hilly forest areas for many migrating people has affected the LULC type. Because of the refugee camps, forests decreased by about 9.58%, and settlements increased by about 8.25%. However, even after two years of immigration, the loss of vegetation remained dramatic, mainly due to humans' forest encroachment. According to reports, the Rohingya households are increasingly in need of fuelwood or lumber, which they take from neighboring woods. The Rohingya households currently require 750,000 kg of fuelwood per day [50], enormously pressing surrounding forest resources. According to a study, the migrants have cleared 5013 acres of dense forest so far, and the number is growing [51]. According to this analysis, between 2017 and 2019, 1876 ha of forested land were lost in the Kutupalong and Balukhali regions [6]. used a remote sensing database to map the Teknaf sub-district, where they found 1284.48 ha of vegetation degradation after just 4 months of the present influx. Using the RF categorization method [2], determined that the whole camp lost 5650 wooded acres between December 2016 and February 2017. Vegetation loss and forest clearance have put a lot of pressure on the ecosystems of Teknaf Wildlife Sanctuary, Himchari National Park, and Inani National Park and their neighboring buffer zones. It has been discovered [52] that numerous species of both plants and animals that are considered vital or endangered call these forest preserves home. These crucial ecosystems are severely disturbed by the refugees' human activities. Having their corridors and routes disturbed by refugee camps and a shortage of food due to deforestation have led to the species being declared endangered [24]. Human-animal conflict is another risk that increases as forests are encroached upon. The current inflow has resulted in 13 fatalities and over 50 injuries to humans due to man-elephant confrontations [24]. And when the hills lose their natural setting due to deforestation and slope cutting, the risk of landslides increases [53]. However, deforestation has led to a 1 °C rise in land surface temperature. The LST at Kutupalong-Balukhali and the surrounding region has risen by 1.3° Celsius between 2017 and 2019, according to a study by Ref. [3].

The LULC change affects the functionality of ES and causes changes to the ESV. Refugees have converted the forest land into settlements, which has caused the decline of ESV. The total ESV was US $67.83 million in 2017 and US $67.78 million in 2021, owing to a rapid increase in settlements (primarily Rohingya refugee camps). Between 2017 and 2021, the timber industry is predicted to lose US$5.33 million (or 21.97%) in economic surplus from ESV. There may be ramifications for the local biota if the ESV for woodlands undergoes such a drastic shift. Deforestation also increases the risk of natural catastrophes in the region of study (for example, landslides, cyclones, flash floods, and so on). During the course of the investigation, there was a rise in the ESV for regulatory purposes. There were downward trends in the three areas of support, provisioning, and culture during the course of the research. Forest loss reduces the expected survival value of these processes. During the years 2017–2021, twelve ESV sub functions were lowered (i.e., food production, raw materials, climate regulation, disturbance regulation, waste treatment, soil-formation and retention, nutrient cycling, erosion control, biodiversity, genetic resources, recreation and tourism, and cultural). There were growth rates of 12.35%, 12.17%, and 1.21% in water regulation, water supply, and pollination, respectively, between 2017 and 2021.

The vast Rohingya refugees fought for a place to live and harmed natural resources in Bangladesh’s southern hilly region. Natural features (i.e., forest cover, vegetation, waterbodies, hills, etc.) of the Cox’s Bazar area are affected by the refugee influx. It is estimated that the severity of natural feature loss will threaten Bangladesh in the long term. Table 6 summarized how migration impact on environmental and natural resources.

**Table 6:** Statement on the environmental and natural resources degradation due to migration.

Authors	Published year	Statement regarding the environmental and natural resources degradation
[2]	2018	The forest cover around the three camps was degraded by 2283 ha due to the quick increase in refugee camps.
[13]	2020	Estimates of aboveground biomass as well as carbon stock showed a consistent and considerable decline during 2017–2019.
[14]	2021	In the Kutupalong RC including Kutupalong extension camp, out of 27.76% of settlements, 0.35% of settlements, and 9.61% of settlements seem to be at risk of landslide and flood, respectively.
[24]	2019	Human-elephant confrontations occurred due to the forest demolished by the Rohingya camps.
[22]	2021	The socioeconomic and environment status of host community has degraded due to Rohingya influx.
[16]	2022	Near the Rohingya refugee camps, shallow forests have taken the place of the deep forests.
[3]	2020	The LST in the Rohingya camp area climbed dramatically, reaching a maximum of 34 °C, much above the pre-influx level.
[54]	2021	A net loss of 6.6 square miles of forest land has been recorded in the Ukhia Forest area due to the Rohingya influx from 2014 to 2018.

In Bangladesh, protecting, conserving, and managing forest resources sustainably is a daunting task [55]. Numerous reasons have contributed to the deteriorating state of the country’s forest resources, chief among them the fact that many poor and oppressed people rely heavily on the forest for their survival [56]. The current pace of deforestation appears to have been increased by the government’s assistance to refugees fleeing violence in a neighboring country, especially in an ecologically vulnerable ecosystem zone [57]. This research supports global findings that migrants may have a significant impact on local ecosystems, with extensive deforestation being the primary outcome [58]. Although Bangladesh has made many attempts to recover its stolen natural resources, the trend is likely to persist [59]. Additional loss of forest cover would be bad for the ecosystem and might lead to repeated slope collapses, placing Rohingya and local inhabitants at risk of landslides since the research location is prone to landslides [30]. Concerns, such as social tension between the two competing groups, are regularly noted since the Rohingya population in the study area (now 932,940) outnumbers the indigenous population (now 471,768; (Bangladesh Bureau of Statistics, 2011)). We hope that the findings of this research will aid in the creation of strategies for the conservation and management of a forest ecosystem in a particularly ecologically delicate area. In light of these findings, the Bangladeshi government and its development partners may make ecosystem management in the study area a top priority in order to promote ecological sustainability. Some basic recommendations to minimize the environmental and natural resources degradation are shown in Table 7.

**Table 7:** Basic recommendations to minimize the environmental and natural resources degradation.

Recommendation	Descriptions of recommendation
Sustainable land management	Implement sustainable land management practices to prevent further degradation and deforestation. This can include reforestation efforts, implementing sustainable agriculture practices, and promoting the use of alternative fuel sources to reduce pressure on forests.
Environmental impact assessment	Conduct thorough environmental impact assessments to identify and address the potential risks and impacts of the Rohingya influx on natural resources and ecosystems. This will help in developing targeted mitigation and conservation strategies.
Ecosystem restoration	Develop and implement plans for ecosystem restoration in areas affected by degradation. This can involve restoring forests, wetlands, and other natural habitats, and promoting biodiversity conservation.
Infrastructure planning	Incorporate environmental considerations into the planning and development of infrastructure in the Rohingya refugee camps. This includes proper waste management systems, provision of clean water sources, and minimizing the ecological footprint of the camps.
Collaboration and partnerships	Foster collaboration among relevant stakeholders, including government agencies, international organizations, NGOs, and local communities, to jointly address the environmental challenges posed by the Rohingya influx. This includes sharing resources, knowledge, and expertise to develop and implement effective solutions.

## 5 Conclusion

The present study focused on assessing the environmental and ecosystem service impacts of the Rohingya influx in Teknaf and Ukhania in Cox’s Bazar district. Using three supervised machine learning methods (RF, SVM, and ANN), the study analyzed LULC dynamics. The findings revealed a decline in forest area, leading to a reduction in twelve out of sixteen ecosystem service subfunctions between 2017 and 2021. To estimate the total ecosystem service value, the study employed the coefficient of Costanza et al. (1997), and fuzzy logic models were developed to assess the ecological quality (EQ) conditions for 2017 and 2021. The results indicated a deterioration from very good and good EQ conditions to bad and very bad EQ conditions.

The findings of this study hold significant policy implications for forest conservation and management. Particularly in developing countries like Bangladesh, where marginalized populations heavily rely on forests for their sustenance, the study underscores the necessity for sustainable forest resource management. Moreover, the study raises concerns about potential social tensions between the Rohingya and indigenous communities in the study area. It is crucial for future policies to encompass the environmental impacts of migration on local ecosystems and proactively mitigate any adverse effects.

The study’s novelty lies in its utilization of geospatial data to evaluate the environmental impact of the refugee crisis. However, the absence of cloud-free satellite data posed limitations in accurately depicting changes in forest cover. Future research could consider incorporating microwave data collected during the monsoon season to identify shrub and mixed forest regeneration, which would contribute to forecasting future changes in ecosystem services. In addition to the aforementioned points, it is important to acknowledge that the study’s analysis was limited to two specific time periods: before the Rohingya Refugee Influx and after the Rohingya Refugee Influx. This temporal scope may not capture the full complexity and long-term impacts of the influx on the environment and ecosystem services. To gain a more comprehensive understanding, it is recommended that future research encompasses a broader range of time periods, allowing for a deeper examination of temporal trends and changes.

## Author contribution statement

Showmitra Kumar Sarkar: Conceived and designed the experiments; Performed the experiments; Analyzed and interpreted the data; Contributed reagents, materials, analysis tools or data; Wrote the paper.

Md. Mustafa Saroar: Conceived and designed the experiments; Wrote the paper.

Tanmoy Chakraborty: Performed the experiments; Wrote the paper.

## Data availability statement

Data will be made available on request.

## Additional information

No additional information is available for this paper.

## Declaration of competing interest

The authors declare that they have no known competing financial interests or personal relationships that could have appeared to influence the work reported in this paper
